# circKDM4C enhances bladder cancer invasion and metastasis through miR-200bc-3p/ZEB1 axis

**DOI:** 10.1038/s41420-021-00712-9

**Published:** 2021-11-23

**Authors:** Xueyou Ma, Yufan Ying, Jiazhu Sun, Haiyun Xie, Jiangfeng Li, Liujia He, Weiyu Wang, Shiming Chen, Haixiang Shen, Jiahe Yi, Jindan Luo, Xiao Wang, Xiangyi Zheng, Ben Liu, Liping Xie

**Affiliations:** 1 Department of Urology, the First Affiliated Hospital, Zhejiang University School of Medicine, Hangzhou, 310003 China; 2 Cancer Center, Zhejiang University, Hangzhou, 310058 China

**Keywords:** Cell invasion, Epithelial-mesenchymal transition, Bladder cancer

## Abstract

Circular RNAs (circRNAs) play essential roles in human bladder cancer (BCa) development, however, unusual expression patterns and functional dysfunction of circRNAs in BCa have not been evaluated. In this study, we validated that circKDM4C (hsa_circ_0001839), derived from the KDM4C gene, is elevated in BCa cell lines as well as tissues. Functionally, overexpression of circKDM4C significantly enhances, and silencing of circKDM4C suppresses migration and invasion capabilities of BCa cells. Mechanistically, circKDM4C can directly interact with miR-200b-3p and miR-200c-3p as a miRNA sponge, which enhances the expression of ZEB1 and promotes ﻿mesenchymal phenotype. Conclusively, our findings indicate that circKDM4C may act as a pro-oncogenic factor in BCa invasion and metastasis via the circKDM4C/miR-200bc-3p/ZEB1 axis, which is a potential biomarker or therapeutic target for bladder cancer.

## Introduction

Bladder cancer (BCa) is ﻿the most prevalent malignant tumor in the urinary system. Globally, it is the 10^th^ most commonly clinically diagnosed cancer, with about 573,000 new cases and 213,000 deaths in 2020 [1]. Since the morbidity and mortality are higher in males, around four times those among women [2], the disease ranks the 6^th^ most prevalent cancer and the 9^th^ leading cause of cancer-associated mortalities among males [1]. Approximately 70% of newly diagnosed individuals present with non-﻿muscle-invasive BCa (NMIBC), while 30% present with muscle-invasive BCa (MIBC) [3]. The patients with NMIBC tend to relapse and progress to MIBC, even ﻿metastatic disease, which is associated with a poor prognosis. Therefore, elucidation of the mechanisms involved in BCa invasion and metastasis will inform the development of potential therapeutic strategies.

Circular RNAs (circRNAs) comprise a large class of endogenous noncoding RNAs (ncRNAs) that are recognized by covalently closed ﻿continuous loop structures without 5′ caps nor 3′ polyadenylated tails [4]. In the last several years, circRNA-specific bioinformatics algorithms and high-throughput RNA sequencing (RNA-seq) have discovered and identified thousands of circRNAs [5], and found that they are usually abundant, stable, conserved, and with tissue or developmental-stage-specific expression patterns [6, 7]. Different from linear RNAs, circRNAs are much more stable and exonuclease resistant [8]. Numerous researches have reported that circRNAs are dysregulated in various human diseases [9–11], and play indispensable roles in regulating gene expression via sponging miRNAs [12, 13]. ﻿Recent studies have identified several functional circRNAs in BCa, such as ﻿circMYLK, circBCRC3, circ000136, circFAM114A2, circST6GALNAC6, and circFNDC3B [14–19]. However, there are more dysregulated circRNAs in BCa that remain to be investigated and clarified the potential mechanisms and biological functions associated with BCa progression.

In the present study, we identified a KDM4C gene-derived circular RNA, termed circKDM4C, in which expression was remarkably elevated in BCa tissues and cell lines. More importantly, we demonstrated that circKDM4C serves as a “sponge” for miR-200bc-3p to increase ZEB1 expression levels and consequently ﻿enhance BCa cell invasion and metastasis. Our results elucidate the mechanisms of circKDM4C in BCa progression.

## Results

### circKDM4C is elevated in BCa, and mainly distributes in the cytoplasm

**Fig. 1:**
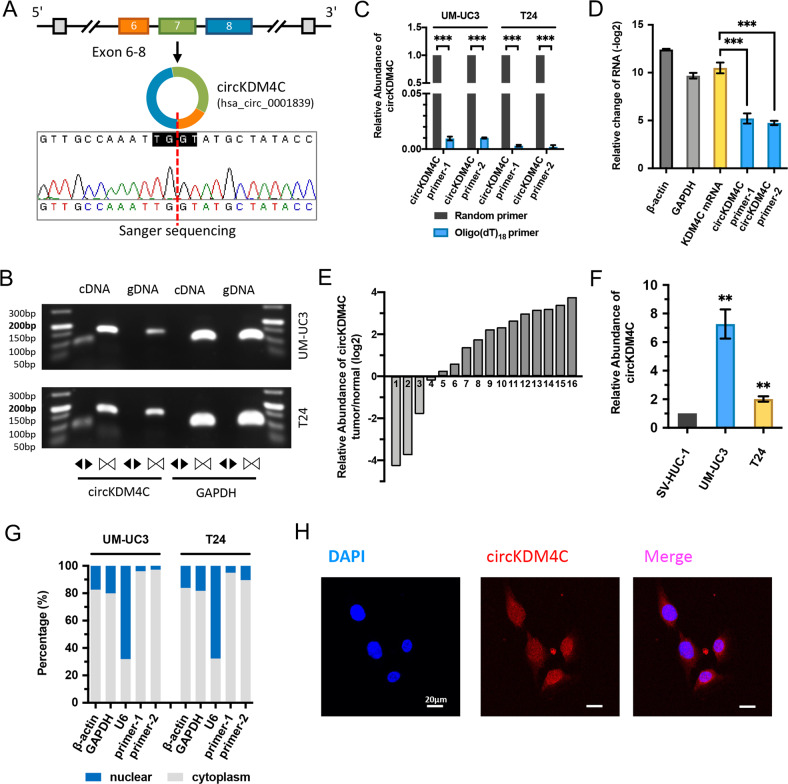
Identification of circKDM4C and its expression in BCa tissues and cell lines. **A** Schematic illustration shows the circularization of KDM4C exon 6–8 formed circKDM4C. Sanger sequencing was used to identify the circKDM4C back-splice junction. Red dashed line indicates the back-splicing site. **B** The existence of circKDM4C was confirmed in UM-UC3 and T24 cells by PCR and agarose-gel electrophoresis. Divergent primers could amplify circKDM4C in cDNA, but not in gDNA. GAPDH (105 bp) was the linear control. circKDM4C, 93 bp; KDM4C mRNA, 158 bp. **C** qRT-PCR analysis of circKDM4C in the cDNA constructed by random primers or oligo-dT primers. circKDM4C level was evaluated by two pairs of divergent primers. **D** circKDM4C and KDM4C mRNA levels ﻿after treatment with or without RNase R in UM-UC3 cells. **E** ﻿The expression of circKDM4C in 16 pairs of BCa tissues and adjacent normal tissues was determined by qRT-PCR. **F** circKDM4C levels in normal uroepithelium cell line (SV-HUC-1) and cancer cell lines (UMUC-3 and T24). **G** circKDM4C levels in nuclear and cytoplasmic fractions of UM-UC3 and T24 cells. **H** ﻿RNA FISH assay showed that circKDM4C was mainly located in the cytoplasm in T24 cells (circKDM4C probe was Cy3-labeled, the nuclei were DAPI-stained). Scale bar = 20 μm. Data are expressed as the means ± SD for *n* = 3. **P < 0.01; ***P < 0.001.

To explore the roles of circRNAs in bladder cancer progression, UM-UC3 and cisplatin-resistant UM-UC3 cells were screened for changes using RNA-seq, which found a lot of circRNAs associated with BCa cisplatin resistance and progression. ﻿Subsequently, we selected fifteen high-abundant circRNAs in UM-UC3 cells and compared their expression levels with human immortalized uroepithelium cells (SV-HUC-1) (Supplementary Table S1, Fig. S1A). On closer examination, circKDM4C (CircBase ID: hsa_circ_0001839) was upregulated both in BCa cell lines and tissues, thus, we decided to follow up and investigate the role of circKDM4C in BCa progression. The human KDM4C gene (GenBank: NM_015061) could generate 43 and 124 different circRNAs according to CircBase [20] and MiOncoCirc [7] database, respectively, and hsa_circ_0001839 consists of the exon 6, 7, and 8 (292 bp) (Fig. 1A). In addition, hsa_circ_0001839 is the most abundant one of all KDM4C gene-derived circRNAs in various tumors shown in MiOncoCirc database (Fig. S1B). First of all, the circular form of circKDM4C was identified. We first designed two pairs of divergent primers for detecting circKDM4C. Then, through Sanger sequencing, we verified the head-to-tail splicing of circKDM4C in the expected-size PCR product by Sanger sequencing (Fig. 1A). However, head-to-tail splicing might be attributed to reverse-transcriptase template switching, trans-splicing, or genomic rearrangements. Therefore, we designed convergent primers for amplifying KDM4C mRNA. The results showed that circKDM4C could only be detected by divergent primers in cDNA, but not in gDNA (Fig. 1B). In addition, circKDM4C was almost undetectable in cDNA constructed using oligo-dT primers, suggesting that circKDM4C was depleted in poly (A)-enriched samples (Fig. 1C). Moreover, circKDM4C was confirmed to be far more resistant to RNase R treatment than linear KDM4C mRNA (Fig. 1D).

To delineate the expression of circKDM4C in BCa, 16 pairs of BCa tissues and paired normal bladder tissues were evaluated by qRT-PCR. circKDM4C was elevated in BCa and tended to be higher in stage T2–T4 and high-grade BCa (Fig. 1E, Fig. S1C, D). Consistently, compared with SV-HUC-1, upregulation of circKDM4C was also confirmed in BCa cell lines (UM-UC3 and T24) (Fig. 1F). The nuclear mass-separation assays and RNA FISH assays were conducted to investigate the intracellular localization of circKDM4C, which was found to be mainly located in the cytoplasm (Fig. 1G, H).

### circKDM4C enhances BCa cell invasion and metastasis in vitro and in vivo

**Fig. 2:**
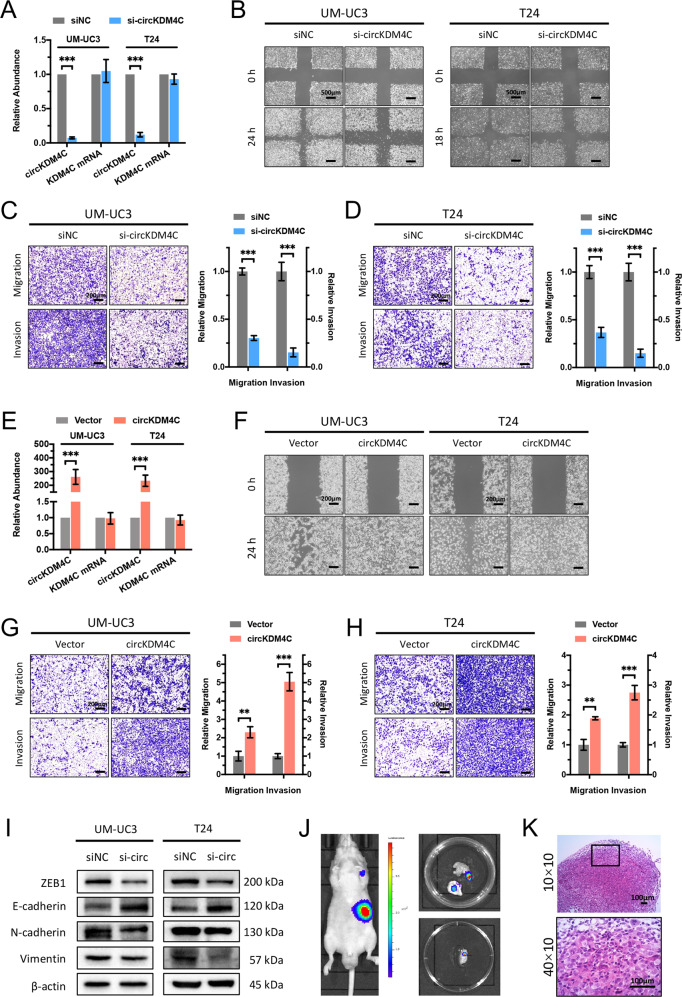
circKDM4C enhances BCa cell invasion and metastasis. **A** circKDM4C and KDM4C mRNA levels in UM-UC3 and T24 cells treated with si-circKDM4C or negative control (siNC). **B** Effects of circKDM4C silencing on cell migration capability were assessed by wound-healing assays. Scale bar = 500 μm. **C, D** Effects of circKDM4C silencing on cell migration and invasion capabilities were determined by transwell migration and Matrigel invasion assays in UM-UC3 (**C**) and T24 (**D**) cells, respectively. Scale bar = 200 μm. **E** circKDM4C and KDM4C mRNA levels in UM-UC3 and T24 cells after transfection of circKDM4C-overexpression plasmid or the corresponding vector plasmids. **F** Effects of circKDM4C overexpression on cell migration capability were assessed by wound-healing assays. Scale bar = 200 μm. **G, H** Effects of circKDM4C overexpression on cell migration and invasion capabilities were determined by transwell migration and Matrigel invasion assays in UM-UC3 (**G**) and T24 (**H**) cells. Scale bar = 200 μm. **I** Effects of circKDM4C silencing on expression levels of EMT-associated proteins were detected by western blotting analysis. **J** Live imaging showed metastasis focus on forelimb (top) and perisplenic lymph node (bottom). **K** H&E staining of perisplenic lymph-node metastasis focus. Scale bar = 100 μm. Data are expressed as the means ± SD for *n* = 3. **P < 0.01; ***P < 0.001.

To investigate the role of circKDM4C in BCa progression, loss-of-function as well as gain-of-function assays were conducted. First, we designed siRNA targeting the back-splice junction of circKDM4C and confirmed the efficiency of circKDM4C silencing using qRT-PCR, while there was no significant effect on linear KDM4C mRNA expression (Fig. 2A). Subsequently, wound-healing and transwell migration assays showed that knockdown of circKDM4C remarkably suppressed cell migration ability (Fig. 2B–D). Similarly, transwell Matrigel invasion assays revealed that knockdown of circKDM4C significantly inhibited cell invasion ability (Fig. 2C, D). To further confirm the role of circKDM4C in BCa, we successfully constructed the circKDM4C-overexpression plasmid, and no remarkable effect in linear KDM4C mRNA was detected (Fig. 2E). Consistently, wound-healing and transwell assays showed that overexpression of circKDM4C remarkably increased migration and invasion capacities of BCa cells (Fig. 2F–H). We further demonstrated that silencing of circKDM4C apparently upregulated the expression levels of epithelial marker, E-Cadherin (*CDH1*), while downregulated ﻿mesenchymal markers, N-Cadherin (*CDH2*) and vimentin (Fig. 2I). Furthermore, we stably infected UM-UC3 cells with shRNA vectors. Assessment of in vivo metastatic ability of BCa cells was performed using nude mice caudal vein metastasis models. Tumor metastases were evaluated six weeks after caudal vein injection of UM-UC3 cells. The results of live imaging (Fig. 2J) and H&E staining (Fig. 2K) demonstrated that circKDM4C silencing significantly reduced metastasis focus. ﻿Therefore, these data indicate that circKDM4C plays an pro-oncogenic role in enhancing BCa cell invasion as well as metastasis.

### circKDM4C serves as a sponge for miR-200b-3p/miR-200c-3p in BCa cells

**Fig. 3:**
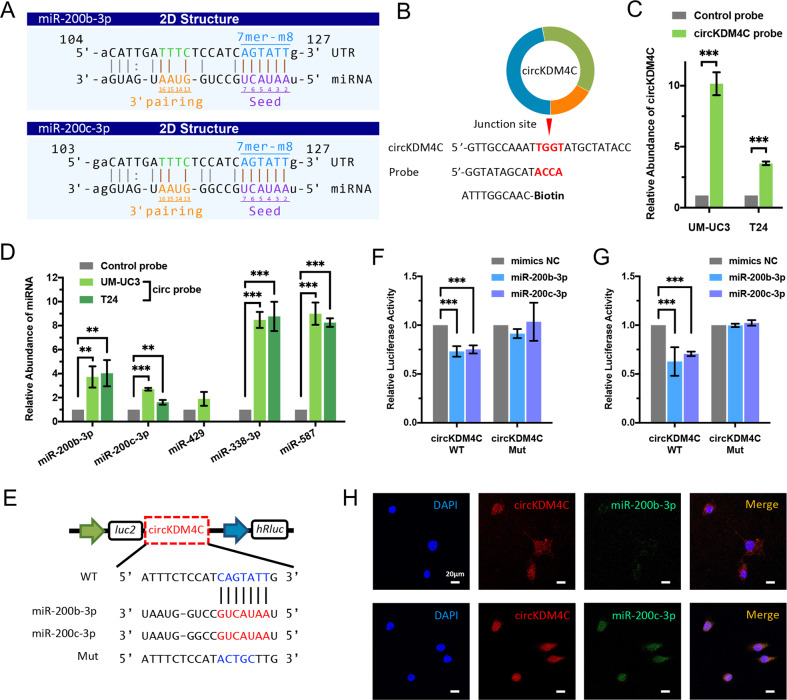
circKDM4C serves as a sponge for miR-200b-3p and miR-200c-3p in BCa cells. **A** MiR-200b-3p/miR-200c-3p binding sites on circKDM4C predicted by TargetScan and miRanda. **B** ﻿Schematic illustration of the specific probe for circKDM4C. circKDM4C probe is complementary with the back-splice junction of circKDM4C. **C** circKDM4C levels in UM-UC3 and T24 lysates after circRNA pulldown using circKDM4C probe or oligo-control probe. **D** Relative enrichments of candidate miRNAs in UM-UC3 and T24 cell lysates. **E** Schematic presentation of circKDM4C-WT and circKDM4C-Mut dual-luciferase reporter vectors. **F, G** Dual-luciferase reporter assays in UM-UC3 (**F**) and T24 (**G**) cells cotransfected with miR-200b-3p/miR-200c-3p mimics and circKDM4C-WT or circKDM4C-Mut vectors, respectively. **H** RNA FISH assay revealed the colocalization of circKDM4C and miR-200b-3p/miR-200c-3p in the cytoplasm in T24 cells (circKDM4C probe was Cy3-labeled, miRNA probes were FITC-labeled, and the nuclei were DAPI-stained). Scale bar = 20 μm. Data are expressed as the means ± SD for *n* = 3. **P < 0.01; ***P < 0.001.

Emerging evidence showed that circRNAs can serve as “miRNA sponge” in cancer cells [12]. CircKDM4C is predominantly distributed in the cytoplasm, implying that it may function post transcriptionally. Therefore, we evaluated circRNA–miRNA interactions by Arraystar’s prediction tools (KangChen, Shanghai, China) based on TargetScan and miRanda [21, 22]. Shown in the prediction, circKDM4C possesses the complementary sequence to miR-200b-3p/miR-200c-3p seed regions (Fig. 3A). Subsequently, we designed the specific biotin-labeled probe for circKDM4C (Fig. 3B) and conducted circRNA pull-down assays to verify whether circKDM4C can directly bind the candidate miRNAs. The specific probe was validated to enrich circKDM4C in UM-UC3 and T24 cell lines (Fig. 3C). Then, the relative enrichment levels of the miR-200c family and the other two miRNAs were assessed. MiR-200b-3p, miR-200c-3p, miR-338-3p, and miR-587 were found to be abundantly enriched by circKDM4C (Fig. 3D). Finally, we chose miR-200b-3p/miR-200c-3p for further study according to the results of functional validation (Fig. S2A).

Then, we constructed dual-luciferase reporters inserted with either the wild-type circKDM4C sequence (WT) or the sequence with mutated binding regions of miR-200b-3p/miR-200c-3p (Mut) (Fig. 3E). Dual-luciferase reporter assay revealed that elevated miR-200b-3p or miR-200c-3p could apparently suppress the luciferase activity of circKDM4C-WT reporter but not that of the mutant (Fig. 3F, G), which indicated that miR-200b-3p and miR-200c-3p could interact with circKDM4C via complementary seed regions. In addition, the RNA FISH assay showed that circKDM4C and miR-200b-3p or miR-200c-3p were colocalized in the cytoplasm (Fig. 3H). Nevertheless, overexpression or silencing of circKDM4C did not significantly influence miR-200b-3p or miR-200c-3p levels in BCa cells (data were not shown). Collectively, the above results suggest that circKDM4C can directly bind miR-200b-3p and miR-200c-3p in BCa cells.

### miR-200b-3p and miR-200c-3p suppress BCa cell invasion and metastasis via targeting ZEB1

**Fig. 4:**
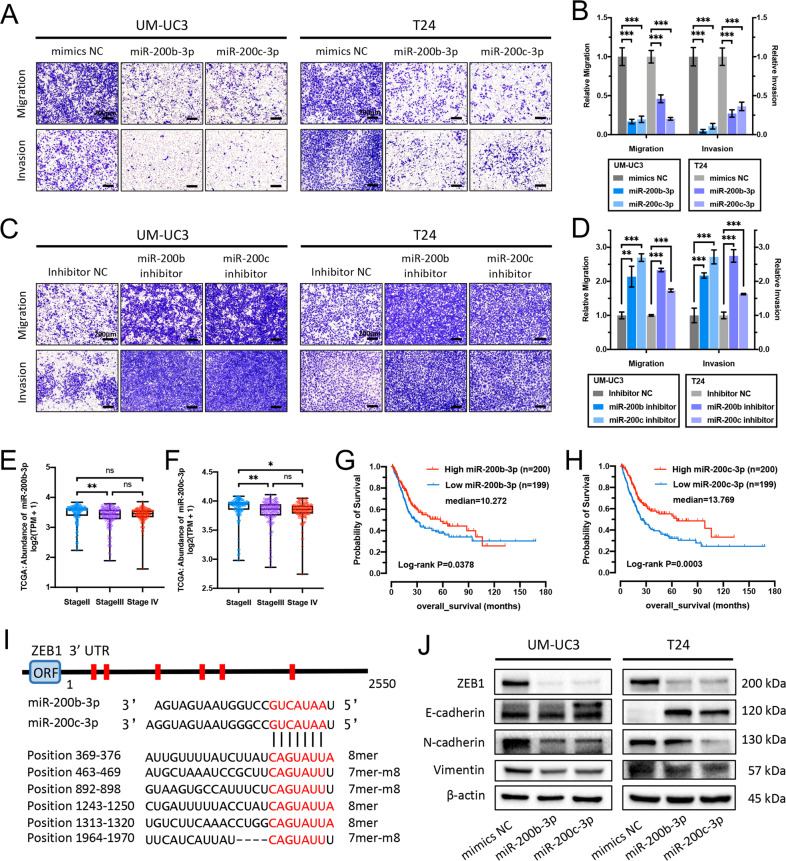
miR-200b-3p and miR-200c-3p suppress BCa cell invasion and metastasis via targeting ZEB1. **A–D** Effects of miR-200b-3p/miR-200c-3p mimics (**A, B**) and miR-200b-3p/miR-200c-3p inhibitors (**C, D**) on cell migration and invasion capabilities were assessed through transwell migration and Matrigel invasion assays in UM-UC3 and T24 cells, respectively. Scale bar = 200 μm. **E, F﻿** MiR-200b-3p and miR-200c-3p expression levels in different pathologic-stage BCa from the TCGA database. **G, H** Kaplan–Meier survival analysis indicated better survival probability in BCa patients with higher miR-200b-3p (**G**) or miR-200c-3p (**H**) expression levels (TCGA database). **I** Schematic graph of human ZEB1 3′ UTR and conserved miR-200bc-3p-site sequences. ORF: open-reading frame. **J** Western blotting assays indicated that miR-200b-3p/miR-200c-3p can downregulate the expressions of ZEB1, N-Cadherin, and vimentin, while upregulating E-Cadherin in BCa cells. Data are expressed as the means ± SD for *n* = 3. ns, no significance; *P < 0.05; **P < 0.01; ***P < 0.001.

MiR-200 superfamily members ﻿play vital roles in ﻿epithelial-to-mesenchymal transition (EMT) of many epithelial tumors, including human bladder cancer [23, 24]. Moreover, the miR-200bc-3p-﻿ZEB1﻿ axis is crucial in regulating this process [25]. Consistent with this, treating UM-UC3 and T24 cells with miR-200b-3p/miR-200c-3p mimics apparently suppressed cell migration and invasion abilities assessed by transwell assays (Fig. 4A, B) and wound-healing assays (Fig. S2B). In contrast, transfection of miR-200b-3p/miR-200c-3p inhibitor remarkably improved migration and invasion capabilities of BCa cells (Fig. 4C, D, Fig. S2B, C). ﻿﻿Furthermore, miR-200b-3p and miR-200c-3p expression levels in BCa tissues were analyzed in 405 patients of TCGA project [26], and the results showed a negative correlation between miR-200b-3p or miR-200c-3p levels and pathologic stages (Fig. 4E, F). ﻿Kaplan–Meier survival analysis showed better survival probability in BCa patients with higher expression levels of miR-200b-3p or miR-200c-3p (Fig. 4G, H). According to the prediction of TargetScan and miRDB [27] database, the ZEB1 gene harbors six conserved miR-200bc-3p sites in its 3′ UTR (Fig. 4I). Coexpression analysis based on 405 bladder cancer samples of TCGA project revealed the apparently negative correlation between expressions of ZEB1 and miR-200b-3p or miR-200c-3p (Fig. S2D, E). In addition, western blotting assays demonstrated that miR-200b-3p and miR-200c-3p mimics observably inhibited ZEB1, N-Cadherin (*CDH2*), and vimentin expressions, while increased E-Cadherin (*CDH1*) expression (Fig. 4J). ﻿Taken together, these results emphasize the significance of the miR-200bc-3p-﻿ZEB1 axis in the invasion and metastasis of BCa cells.

### circKDM4C regulates ZEB1 expression and enhances BCa progression via sponging miR-200b-3p and miR-200c-3p

**Fig. 5:**
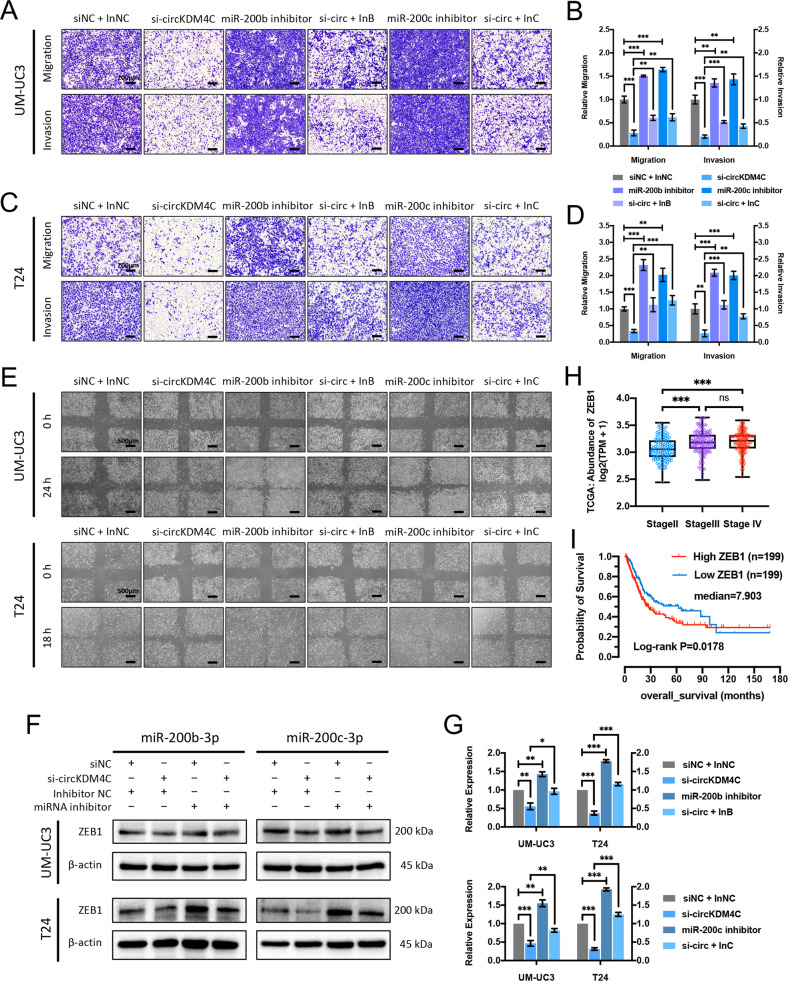
circKDM4C sponges miR-200b-3p and miR-200c-3p to enhance ZEB1 expression and promote BCa cell migration and invasion. **A–D** Transwell migration and Matrigel invasion assays for UM-UC3 (**A, B**) and T24 (**C, D**) cells after circKDM4C knockdown alone or cotransfected with miR-200b-3p or miR-200c-3p inhibitors. Scale bar = 200 μm. **E** Wound-healing assays of UM-UC3 and T24 cells. Scale bar = 500 μm. **F** Western blotting assays showed that miR-200b-3p/miR-200c-3p inhibitors partly reversed the decrease of ZEB1 expression induced by circKDM4C knockdown in BCa cells. **G** Quantification of ZEB1 levels. **H** Expression levels of ZEB1 in different pathologic-stage BCa from the TCGA database. **I** Kaplan–Meier survival analysis indicated poorer survival probability in BCa patients with higher ZEB1 expression. InNC: inhibitor NC; InB/InC: miR-200b-3p/miR-200c-3p inhibitor. Data are expressed as the means ± SD for *n* = 3. ns, no significance; **P < 0.01; ***P < 0.001.

**Fig. 6:**
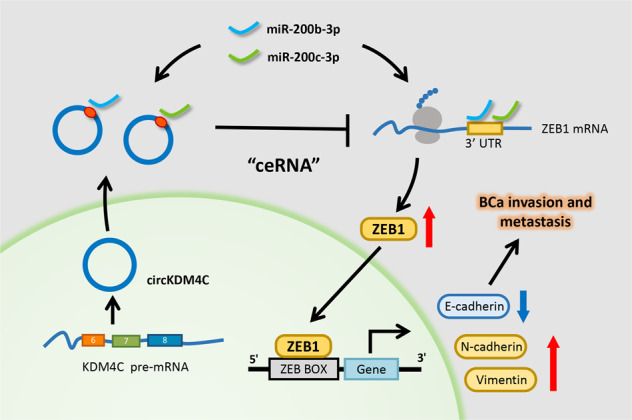
Schematic presentation of the circKDM4C-regulated pathway in BCa cells. circKDM4C derives from the KDM4C gene and consists of the exon 6–8. circKDM4C possesses miR-200bc-3p binding site and could directly sponge miR-200b-3p/miR-200c-3p to indirectly increase ZEB1 expression, which promotes bladder cancer invasion and metastasis eventually.

To further investigate whether circKDM4C promotes BCa progression via sponging miR-200b-3p and miR-200c-3p, “rescue” experiments were conducted to evaluate the functional interactions of circKDM4C and miR-200b-3p/miR-200c-3p. Transwell assays demonstrated that knockdown of circKDM4C led to inhibition of migration and invasion abilities, ﻿whereas transfecting miR-200b-3p or miR-200c-3p inhibitors could partly attenuate this effect (Fig. 5A–D). Similar results were also observed in the wound-healing assays (Fig. 5E). Consistently, ﻿western blotting assays revealed that circKDM4C silencing-induced suppression of ZEB1 can be partly reversed by ectopic miR-200b-3p or miR-200c-3p inhibitors in BCa cells (Fig. 5F, G). In addition, a positive correlation between the ZEB1 levels and pathologic stages of 405 BCa patients from TCGA project was established (Fig. 5H). Kaplan–Meier survival analysis showed poorer survival probability in BCa patients with higher ZEB1 expressions (Fig. 5I). ﻿In summary, these data indicate that circKDM4C could ﻿function as a sponge for miR-200b-3p/miR-200c-3p to increase ZEB1 expression, which promotes bladder cancer progression eventually (Fig. 6).

## Discussion

Circular RNAs (circRNAs), which form a large family of endogenous ncRNAs, are characterized by covalently closed loops. In the 1970s, circRNAs were ﻿originally observed in RNA viruses [28]. Subsequently, high-throughput RNA-seq and bioinformatics algorithms showed that circRNAs are extensively expressed in several mammalian cell lines and across diverse species [9]. Previous researches have demonstrated that expressions of circRNAs are cell-type specific and tissue or developmental-stage specific, which implied their potential relationships with the origin and development of disease [4, 11]. Emerging evidence has demonstrated that circRNAs could serve as oncogenic factors or tumor suppressors to affect oncogenesis and tumor development. ﻿﻿Recently, several dysregulated circRNAs have been identified in BCa development, these studies showed that circRNAs could influence associated pathways to regulate the cell cycle, cell migration and invasion, angiogenesis, as well as cisplatin chemoresistance [29–31]. In our work, we validated that circKDM4C was elevated in BCa tissues and cell lines, and tended to be positively correlated with muscle invasion and ﻿histological grade of BCa. Moreover, we demonstrated that circKDM4C enhanced BCa cell invasion and metastasis by in vitro and in vivo studies. Therefore, circKDM4C is a potential pro-oncogenic factor for BCa progression and could be applied as a novel potential biomarker or therapeutic target for BCa management.

The “miRNA sponge” model, also referred to as competing endogenous RNA (ceRNA), is one of the most classical mechanisms for ncRNAs, including circRNAs [12, 13]. Previous studies demonstrated that circRNAs functioned as miRNA sponges should derive from protein-encoding exons and predominantly locate in the cytoplasm [32]. Since circKDM4C is derived from three exons of the KDM4C gene and mainly localized in the cytoplasm, we performed circRNA RNA pull-down and dual-luciferase reporter assays. MiR-200b-3p and miR-200c-3p were found to be pulled down with direct interactions by circKDM4C. Furthermore, following functional ﻿assays revealed that miR-200b-3p and miR-200c-3p could suppress BCa cell migration and invasion, and partly reverse the effects of circKDM4C in BCa cells. Actually, our data clarified that circKDM4C could sponge miR-200b-3p and miR-200c-3p as ceRNA in BCa cells.

Epithelial-to-mesenchymal transition (EMT) is a well-described mechanism for cancer progression and metastasis [33]. Our previous studies have confirmed several functional miRNAs regulating EMT process in BCa, including miR-323a-3p [34], miR-381-3p [35], and miR-502-5p [36]. The miR-200 superfamily members have been validated to be crucial in regulating EMT process [24, 37]. ﻿The miR-200-ZEB1 axis was widely investigated in many cancers, including human bladder cancer [38]. ZEB1 (﻿﻿also known as TCF8 and DeltaEF1), which belongs to E-box-binding transcription factors, is an important transcription repressor of ﻿*CDH1* (encoding E-cadherin) and crucial in the complex network of transcription repressors ﻿that regulate EMT program [39, 40]. A recent research indicates that the miR-200c family (﻿miR-200b, -200c, and -429) and not the coexpressed miR-141 family (﻿miR-200a, -141) is responsible for regulation of ZEB1 and EMT [25]. ﻿Consistently, we revealed that overexpression and inhibition of miR-200b-3p or miR-200c-3p could reduce or enhance migration and invasion capabilities of BCa cells, and be ﻿sufficient to reverse or induce EMT, respectively, which indicated that miR-200b-3p and miR-200c-3p functioned as tumor suppressors in BCa. In addition, we revealed that miR-200b-3p or miR-200c-3p levels were negatively correlated with BCa pathologic stages, but positively correlated with survival probability in BCa patients, consisting with previous studies [38, 41]. Consistently, silencing of circKDM4C in BCa cells decreases ZEB1 expression, which was partly reversed by suppressing miR-200b-3p or miR-200c-3p. In summary, the miR-200bc-3p-ZEB1 axis plays a critical role in circKDM4C-mediated pro-oncogenic functions in BCa. This study provides further evidence for the post-transcriptional regulation of ZEB1 by circRNA and miRNA in BCa.

Moreover, silencing of circKDM4C inhibited BCa cell proliferation and induced apoptosis, which was not mediated by miR-200b-3p or miR-200c-3p. The detailed molecular mechanism needs further investigation. Interestingly, circKDM4C (hsa_circ_0001839) has been reported as a tumor suppressor in breast cancer [42] and acute myeloid leukemia (AML) [43]. These results also indicate that circKDM4C may function via other miRNAs and involve in biological processes not tested in our present work to regulate developments of BCa and other tumors in particular stage.

In conclusion, circKDM4C is remarkably elevated in BCa cell lines and tissues, and it promotes BCa invasion and metastasis via directly sponging miR-200b-3p/miR-200c-3p to indirectly increase ZEB1 expression (Fig. 6). Our results elucidate on the underlying mechanisms of circKDM4C in BCa development, which may provide a novel potential biomarker or inform the development of therapeutic strategies for bladder cancer.

## Materials and methods

### Patient tissue specimens

BCa tissues and the paired adjacent normal tissues were acquired from patients who had been subjected to transurethral resection of bladder tumors or radical cystectomy at the First Affiliated Hospital, Zhejiang University School of Medicine (Hangzhou, China). ﻿﻿Immediately, tissue specimens were frozen in liquid nitrogen and then stored at -80 °C for RNA extraction. The Ethics Committee of the First Affiliated Hospital, Zhejiang University School of Medicine, approved this study. All patients provided written informed consent. Detailed clinical and pathological features were shown in Supplementary Table S2.

### Cell lines and cell culture

All cell lines (UM-UC3, T24, and SV-HUC-1) were acquired from the Cell Bank of Type Culture Collection of the Chinese Academy of Sciences (Shanghai, China) ﻿with authentication via short tandem-repeat DNA profiling. Culturing of UM-UC3 cells was done in Minimum Essential Medium (MEM, Corning, USA), T24 cells in RPMI 1640 medium (BI, Israel), and SV-HUC-1 cells in F-12K medium (Gibco, USA) with 10% fetal bovine serum (FBS, BI) at 37 °C and 5% CO_2_ in humidified environment.

### RNA extraction, RNA-seq, RNase-R treatment, and qRT-PCR

Genomic DNA (gDNA) was extracted using DNA Kit (BioTeke, China). Total RNAs from cells and tissues were isolated with ﻿RNAiso plus (Takara, Japan). RNA-seq and circRNA-specific bioinformatics algorithms were performed by RiboBio (Guangzhou, China). Nuclear and cytoplasmic fractions were separated using the PARIS Kit (Life Technologies, USA). Total RNA was incubated at 37 °C for 15 min with 2 U/mg of RNase R (Epicenter, USA). Reverse transcriptions were proceeded with PrimeScript RT Master Mix (Takara) or Mir-X miRNA First-Strand Synthesis Kit (Takara), and quantitative real-time PCR (qRT-PCR) was conducted by TB Green Premix Ex Taq II (Takara) in CFX96 system (Bio-Rad, USA). β-actin and U6 were used as the internal references. All primers were listed in Supplementary Table S3.

### Sanger sequencing and nucleic acid electrophoresis

gDNA and cDNA templates were amplified with ﻿Platinum Taq DNA Polymerase (Thermo Fisher, USA) for 40 cycles. The electrophoresis was conducted in 2% GelRed (BioSharp, Hefei, China) stained agarose gel, and the bands were observed under UV radiation. The PCR products were sent to TsingKe (Hangzhou, China) for Sanger sequencing.

### Plasmid construction and transfection

siRNAs and miRNA mimics/inhibitors were synthesized by RiboBio (China). ﻿Human circKDM4C cDNA was cloned into pcDNA3.1(+) CircRNA Mini Vector (Addgene, USA) to construct the circKDM4C-overexpression plasmid. shRNA targeting circKDM4C was designed based on siRNA sequences and cloned into the psi-LVRU6P/Puro vector (GeneCopoeia, USA). Transfection of siRNAs and miRNA mimics/inhibitors into cells was done using the jetPRIME transfection reagent (Polyplus, USA). Moreover, plasmid transfection was done using the FuGENE HD transfection reagent (Promega, USA) based on the manufacturer’s protocol. The corresponding sequences were listed in Supplementary Table S3.

### RNA fluorescence in situ hybridization (FISH)

The Cy3-labeled circKDM4C and FITC-labeled miR-200b-3p and miR-200c-3p probes were synthesized by RiboBio (China). Hybridization was performed using the Fluorescent in Situ Hybridization Kit (RiboBio). Fluorescence imaging was conducted by confocal microscopy (Olympus, Japan). The sequences of the probes were listed in Supplementary Table S3.

### Wound-healing and transwell assays

In wound-healing assays, 200-μL pipette tips were used to make wounds in the center of the 12-well plates. Subsequently, serum-free media were used for cell culture. The cell migration process was observed under phase-contrast microscope (Olympus) after 18 hours (for T24) or 24 hours (for UM-UC3).

Transwell assays were conducted as previously described [20]. Transwell chambers (﻿Merck Millipore, USA) without Matrigel were used for cell migration assays, and chambers with Matrigel for cell invasion assays. In migration assays, ﻿nearly 4 × 10^4^ T24 cells or 6 × 10^4^ UM-UC3 cells were suspended in 200 μL of serum-free medium and added to the upper chambers. For invasion assays, nearly 7 × 10^4^ T24 cells or 10 × 10^4^ UM-UC3 cells were used. Then, the chambers were placed into a 24-well plate filled with 800 μL of medium containing 10% FBS. ﻿﻿After incubation for 24 h at 37 °C, ﻿the cells on the lower surface were fixed with methanol and stained with 0.1% crystal violet. The images were captured by phase-contrast microscope (Olympus).

### Animal experiments

Approval for the use of animals in this study was granted by the Animal Experimental Ethical Committee of the First Affiliated Hospital, Zhejiang University School of Medicine. Twenty mice were used for the tumormetastasis model. The 2 × 10^6^ stably transfected UM-UC3 cells (shNC, shcircKDM4C) were injected into four-week-old male BALB/c nude mice via caudal vein. After six weeks, bioluminescence of the tumor was detected by living fluorescence imaging system. ﻿Finally, the mice were sacrificed and suspected metastases were fixed and stained with hematoxylin and eosin (H&E).

### circRNA pull-down assay

The specific biotin-labeled circKDM4C probe, which targets the back-splicing junction, was synthesized by RiboBio (China). The circRNA pull-down assay was conducted based on the procedure described in a previous study [15]. Briefly, T24 or UM-UC3 cells transfected with circKDM4C-overexpression plasmid were harvested, lysed, and centrifugated. circKDM4C-specific and control probes were incubated for 2 h at 25 °C in the presence of M-280 streptavidin magnetic beads (Invitrogen, USA) to generate the probe-coated beads. Then, cell lysates were incubated overnight at 4 °C in the presence of the bead–probe complex. ﻿Subsequently, the beads were washed, after which RNA was isolated and purified using the RNeasy Mini Kit (QIAGEN, Germany). The target-gene levels were assessed by qRT-PCR.

### Dual-luciferase reporter assay

Cloning of circKDM4C wild-type (WT) and mutated binding region of miR-200bc-3p (Mut) sequence cDNA into ﻿pmirGLO dual-luciferase reporter vectors (Promega) was validated by DNA sequencing. The UM-UC3 and T24 cells were cultured in 96-well plates and cotransfected with dual-﻿luciferase reporter vectors and NC/miRNA mimics. After 48 h, relative luciferase activities were detected with the dual-luciferase reporter assay kit (Promega) by Berthold Detection System (Germany).

### Western blotting

Western blotting assays were conducted as previously described [44]. Antibodies are recorded: anti-E-cadherin (1:1000; #3195, ﻿Cell Signaling Technology, USA), anti-N-cadherin (1:5000; ﻿66219-1-Ig, Proteintech, UK), anti-Vimentin (1:1000; #5741, CST), and anti-ZEB1 (1:1000; #3396, CST), anti-β-actin (1:3000; #4970, CST), goat anti-mouse or anti-rabbit secondary antibody (1:5000, Proteintech). The blots were visualized using commercial ECL kit (BioSharp). β-actin was the internal reference.

### Statistical analysis

The data are presented as the means ± standard deviation (SD). Differences between groups were determined by Student’s *t*-test or chi-square tests. *P*-value less than 0.05 was regarded as statistical significance.

## Supplementary information

Supplementary Figure legends

Supplementary Figure S1

Supplementary Figure S2

Supplementary Table S1

Supplementary Table S2

Supplementary Table S3

## Data Availability

All data generated or analyzed during this study are included in this article.
