# Supplementary material for: circKDM4C enhances bladder cancer invasion and metastasis through miR-200bc-3p/ZEB1 axis
Source: Cell Death Discov. 2021 Nov 23;7:365. doi: 10.1038/s41420-021-00712-9 (PMC8608878; doi:10.1038/s41420-021-00712-9)
Supplement: Supplementary file 1 — Supplementary Figure legends [file 41420_2021_712_MOESM1_ESM.docx]

**Supplementary Figure legends**

**Figure S1. circKDM4C levels in BCa cell lines and tissues.**

**A** Expression levels of candidate circRNAs in SV-HUC-1 and UM-UC3 cells. **B** Expression levels of circKDM4C (hsa_circ_0001839) in various tumors showed in MiOncoCirc database. BLCA: bladder cancer. **C, D** Expression of circKDM4C in different pathologic stages and histological grades BCa of 16 patients. Data are expressed as the means ± SD for n=3. *P < 0.05; **P <0.01; ***P < 0.001.

**Figure S2. miR-200b-3p and miR-200c-3p suppress BCa cells migration via targeting ZEB1.**

**A** Effects of miR-338-3p and miR-587 on cell migration capability were assessed by transwell migration assays in UM-UC3 and T24 cells, respectively. **B, C** Effects of miR-200b-3p/miR-200c-3p inhibitors on cell migration capability were assessed via wound-healing assays in T24 (**B**) and UM-UC3 (**C**) cells. **D, E** Co-expression evaluation of ZEB1 and miR-200b-3p (**D**) or miR-200c-3p (**E**) levels in 405 bladder cancer samples from TCGA project, Pearson correlation index was -0.4818 (p＜0.00001) and -0.5899 (p＜0.00001), respectively.
