# Supplementary figures and images for: circKDM4C enhances bladder cancer invasion and metastasis through miR-200bc-3p/ZEB1 axis
Source: Cell Death Discov. 2021 Nov 23;7:365. doi: 10.1038/s41420-021-00712-9 (PMC8608878; doi:10.1038/s41420-021-00712-9)

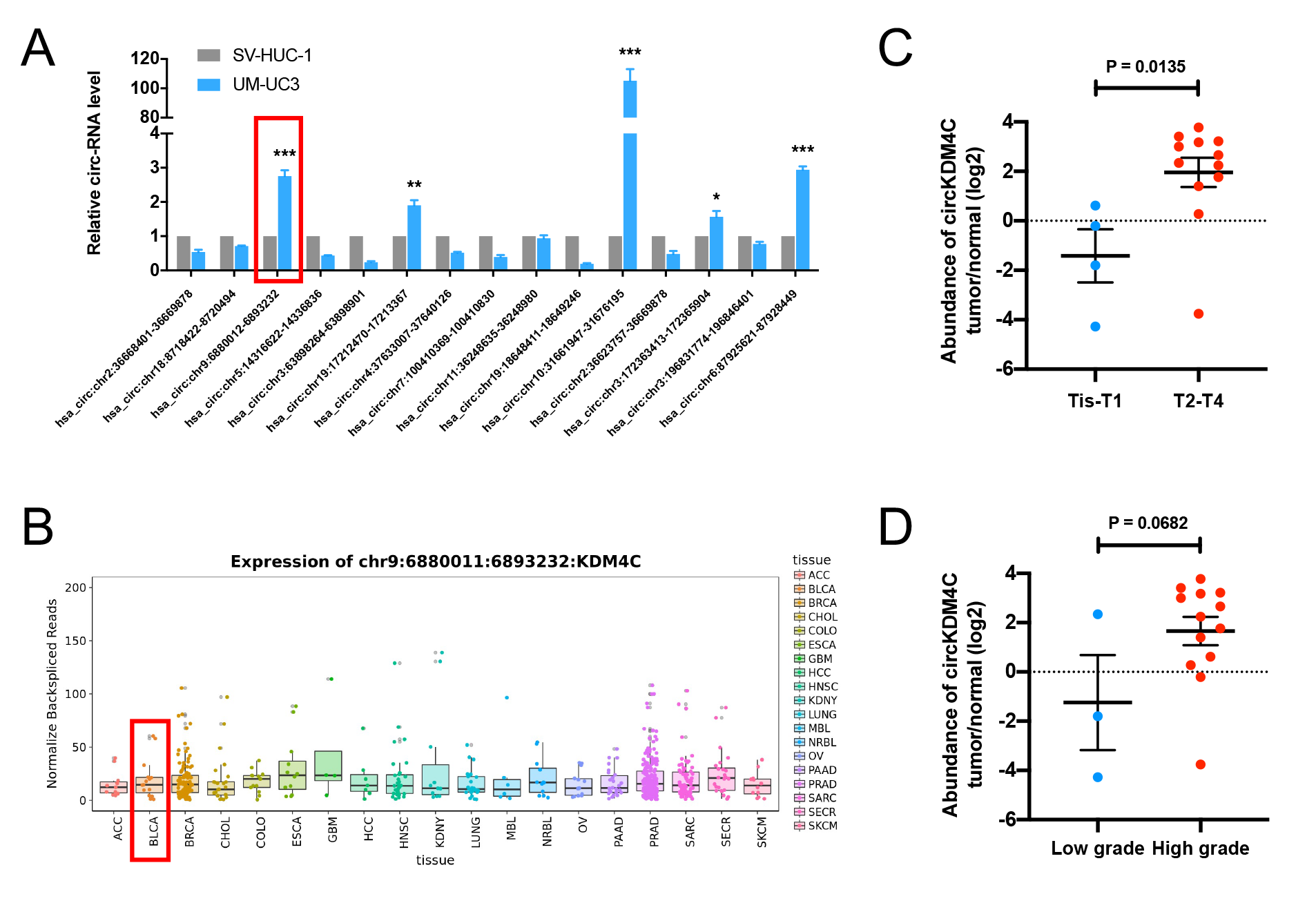

Supplement: Supplementary file 2 — Supplementary Figure S1 [file 41420_2021_712_MOESM2_ESM.tif]

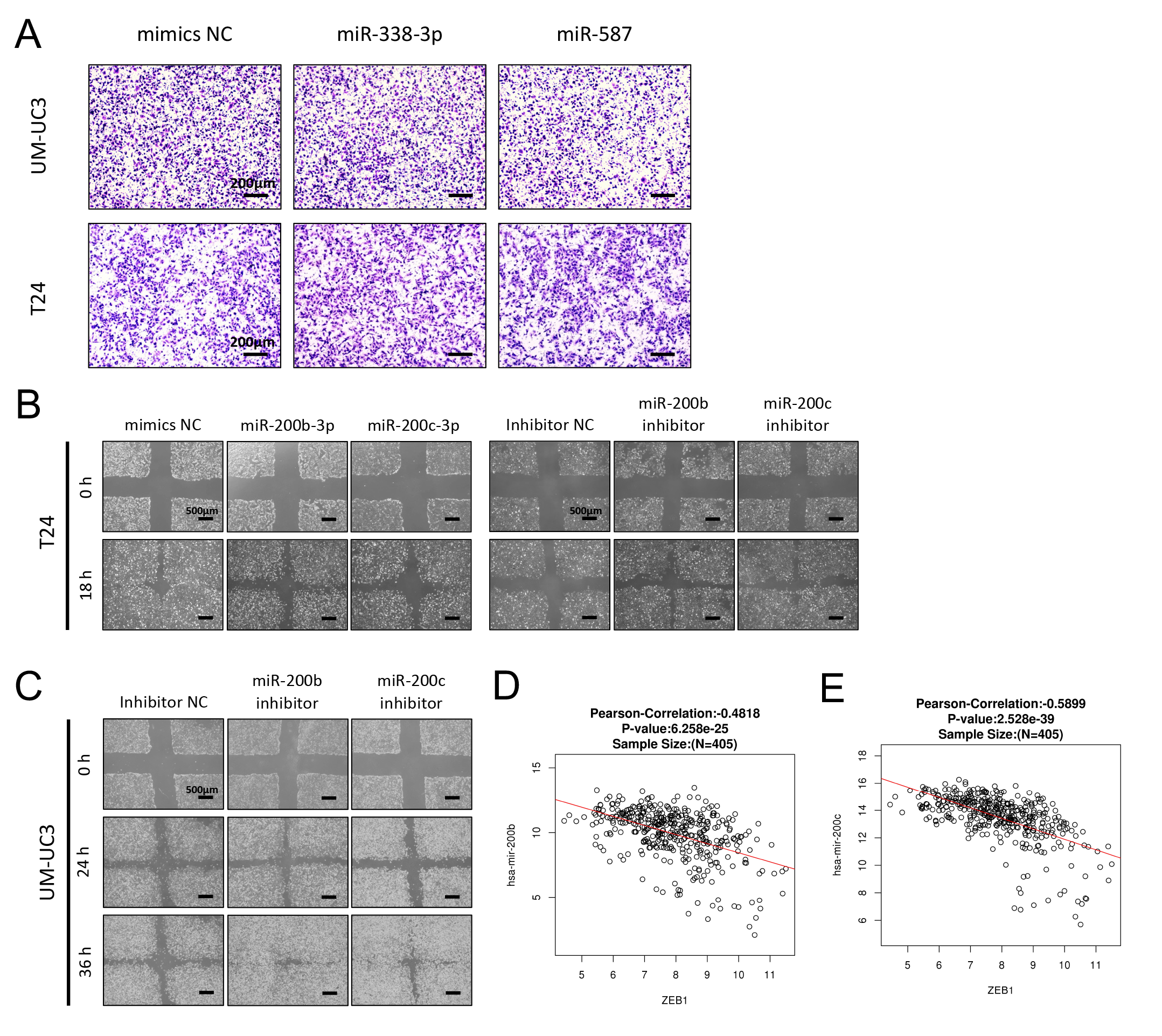

Supplement: Supplementary file 3 — Supplementary Figure S2 [file 41420_2021_712_MOESM3_ESM.tif]
