# Supplementary material for: circKDM4C enhances bladder cancer invasion and metastasis through miR-200bc-3p/ZEB1 axis
Source: Cell Death Discov. 2021 Nov 23;7:365. doi: 10.1038/s41420-021-00712-9 (PMC8608878; doi:10.1038/s41420-021-00712-9)
Supplement: Supplementary file 5 — Supplementary Table S2 [file 41420_2021_712_MOESM5_ESM.docx]

**Table S2. Clinicopathological features of 16 bladder cancer patients and the expressions of circKDM4C.**

| Parameters | Group | Cases | circKDM4C expression | | | | P value |
| --- | --- | --- | --- | --- | --- | --- | --- |
|  |  |  | Low | % | High | % |  |
| Gender | Male | 13 | 6 | 46.2 | 7 | 53.8 | >0.9999 |
|  | Female | 3 | 2 | 66.7 | 1 | 33.3 |  |
| Age at surgery | <55 | 3 | 2 | 66.7 | 1 | 33.3 | >0.9999 |
|  | ≥55 | 13 | 6 | 46.2 | 7 | 53.8 |  |
| Pathological stage | pTis-T1 | 4 | 4 | 100 | 0 | 0 | 0.0833 |
|  | pT2-T4 | 12 | 4 | 33.3 | 8 | 66.7 |  |
| Grade | Low | 3 | 2 | 66.7 | 1 | 33.3 | 0.6888 |
|  | High | 13 | 6 | 65.7 | 7 | 34.3 |  |
| Lymph node metastasis | Absent | 11 | 6 | 54.5 | 5 | 45.5 | >0.9999 |
|  | Present | 5 | 2 | 40.0 | 3 | 60.0 |  |
| Total |  | 16 | 8 | 50.0 | 8 | 50.0 |  |

P < 0.05 represents statistical significance (Chi-square test).
