# Supplementary material for: circKDM4C enhances bladder cancer invasion and metastasis through miR-200bc-3p/ZEB1 axis
Source: Cell Death Discov. 2021 Nov 23;7:365. doi: 10.1038/s41420-021-00712-9 (PMC8608878; doi:10.1038/s41420-021-00712-9)
Supplement: Supplementary file 6 — Supplementary Table S3 [file 41420_2021_712_MOESM6_ESM.docx]

**Table S3. Primers and oligonucleotide sequences.**

| **Primers for PCR (5’-3’)** | | | |
| --- | --- | --- | --- |
| circKDM4C Forward1/divergent F | ﻿TTTGCTACTGTCAGATGGATTGAC |  |  |
| circKDM4C Reverse1/divergent R | ﻿TGAGCTAGTCTTTCAAGTCGTTTTC |  |  |
| circKDM4C Forward2 | ﻿TGGAAAAGTTGCCAAATTGG |  |  |
| circKDM4C Reverse2 | ﻿GGAGCTGCTTGGGAAAAAAC |  |  |
| KDM4C Forward/convergent F | CATGGAGTCTAAAGGAGCCCA |  |  |
| KDM4C Reverse/convergent R | TGTACTGAGTGAACAGTCCTGA |  |  |
| divergent-GAPDH Forward | CGACCACTTTGTCAAGCTCA |  |  |
| divergent-GAPDH Reverse | GACCTTCACCTTCCCCATTT |  |  |
| GAPDH-convergent-Forward | GCCGTCTAGAAAAACCTGCC |  |  |
| GAPDH-convergent-Reverse | CCACCTGGTGCTCAGTGTAG |  |  |
| β-actin Forward | CATGTACGTTGCTATCCAGGC |  |  |
| β-actin Reverse | CTCCTTAATGTCACGCACGAT |  |  |
| miR-200a-3p-Forward | TAACACTGTCTGGTAACGATGT |  |  |
| miR-200b-3p-Forward | TAATACTGCCTGGTAATGATGA |  |  |
| miR-200c-3p-Forward | TAATACTGCCGGGTAATGATGGA |  |  |
| miR-141-3p-Forward | TAACACTGTCTGGTAAAGATGG |  |  |
| miR-429-Forward | TAATACTGTCTGGTAAAACCGT |  |  |
| miR-587-Forward | TTTCCATAGGTGATGAGTCAC |  |  |
| miR-338-3p-Forward | TCCAGCATCAGTGATTTTGTTG |  |  |
| miRNA reverse | Provided by Takara |  |  |
| U6 Forward | Provided by Takara |  |  |
| U6 Reverse | Provided by Takara |  |  |
| **Oligonucleotide (5’-3’)** | | |  |
| siNC | Provided by Ribobio |  |  |
| si-circKDM4C | GCCAAATTGGTATGCTATA |  |  |
| miR-200b-3p mimics | Provided by Ribobio |  |  |
| miR-200c-3p mimics | Provided by Ribobio |  |  |
| miR-200b-3p inhibitor | Provided by Ribobio |  |  |
| miR-200c-3p inhibitor | Provided by Ribobio |  |  |
| circKDM4C pull down probe (biotin) | GGTATAGCATACCAATTTGGCAAC |  |  |
| control probe/sense | GTTGCCAAATTGGTATGCTATACC |  |  |
| **RNA FISH probes (5’-3’)** | | |  |
| control FISH Probe (FITC) | GGCTCTAGAAAAGCCTATGC |  |  |
| circKDM4C-fish probe (Cy3) | AGGTATAGCATACCAATTTGGCAACTTT |  |  |
| miR-200b-3p-fish probe (FITC) | TCATCATTACCAGGCAGTATTA |  |  |
| miR-200c-3p-fish probe (FITC) | TCCATCATTACCCGGCAGTA |  |  |
